# *Caenorhabditis elegans *chromosome arms are anchored to the nuclear membrane via discontinuous association with LEM-2

**DOI:** 10.1186/gb-2010-11-12-r120

**Published:** 2010-12-23

**Authors:** Kohta Ikegami, Thea A Egelhofer, Susan Strome, Jason D Lieb

**Affiliations:** 1 Department of Biology, Carolina Center for Genome Sciences and Lineberger Comprehensive Cancer Center, The University of North Carolina at Chapel Hill, 407 Fordham Hall, Chapel Hill, North Carolina 27599, USA; 2 Department of MCD Biology, University of California Santa Cruz, 1156 High Street, Santa Cruz, California 95064, USA

## Abstract

**Background:**

Although *Caenorhabditis elegans *was the first multicellular organism with a completely sequenced genome, how this genome is arranged within the nucleus is not known.

**Results:**

We determined the genomic regions associated with the nuclear transmembrane protein LEM-2 in mixed-stage *C. elegans *embryos via chromatin immunoprecipitation. Large regions of several megabases on the arms of each autosome were associated with LEM-2. The center of each autosome was mostly free of such interactions, suggesting that they are largely looped out from the nuclear membrane. Only the left end of the X chromosome was associated with the nuclear membrane. At a finer scale, the large membrane-associated domains consisted of smaller subdomains of LEM-2 associations. These subdomains were characterized by high repeat density, low gene density, high levels of H3K27 trimethylation, and silent genes. The subdomains were punctuated by gaps harboring highly active genes. A chromosome arm translocated to a chromosome center retained its association with LEM-2, although there was a slight decrease in association near the fusion point.

**Conclusions:**

Local DNA or chromatin properties are the main determinant of interaction with the nuclear membrane, with position along the chromosome making a minor contribution. Genes in small gaps between LEM-2 associated regions tend to be highly expressed, suggesting that these small gaps are especially amenable to highly efficient transcription. Although our data are derived from an amalgamation of cell types in mixed-stage embryos, the results suggest a model for the spatial arrangement of *C. elegans *chromosomes within the nucleus.

## Background

The nuclear envelope, which consists of nuclear membranes, nuclear pore complexes and the nuclear lamina, primarily functions to separate the nuclear contents from the cytoplasm, and to maintain the structural integrity of the nucleus. However, this barrier is also physically associated with chromatin, which has led to the hypothesis that the nuclear envelope helps to control the spatial arrangement of the genome within the nucleus [1-4]. This three-dimensional organization has increasingly been linked to gene regulatory mechanisms. For example, in multicellular organisms transcriptionally silent, heterochromatic regions are localized close to the nuclear envelope, whereas active regions are more internally localized [1,5]. Therefore, to understand how access to genomic information is regulated, it is crucial to understand how chromosomes are organized spatially within the nucleus.

Interactions between the nuclear envelope and chromosomes have been mapped in fly, mouse, and human cells by recording associations between the genome and B-type lamins and emerin [6-8]. B-type lamins are one of the two major types of lamins in animal cells, and emerin is an inner nuclear transmembrane protein [9]. All of these studies inferred regions of DNA interaction with B-type lamins or emerin using the DamID (DNA adenine methyltransferase identification) technique, in which the proteins are fused with bacterial adenine methyltransferase [6-8,10]. This allows DNA that had interacted with the chimeric protein to be isolated and detected, since adenine methylation does not normally occur in eukaryotic cells. B-type lamin and emerin were found to be associated with large domains up to several megabases in length, which cover about 40% of the genome in mouse and human cells [6,7]. In flies, however, the size and the coverage of lamin-associated regions were not determined precisely because the cDNA microarrays used for detection contained a single probe per gene [8]. Nonetheless, the common finding among human, mouse, and fly is that nuclear envelope-associated regions possess heterochromatic characteristics, such as high levels of histone H3K9 dimethylation and H3K27 trimethylation, low gene density, and low gene expression.

In this study, we identify genomic regions associated with an inner nuclear membrane protein in *Caenorhabditis elegans *utilizing a different approach, chromatin immunoprecipitation (ChIP) of the LEM-2 protein coupled with detection by tiling microarray (ChIP-chip) and next-generation sequencing (ChIP-seq). LEM-2 is a transmembrane protein localized to the inner nuclear membrane, with homologs in a wide variety of organisms, including yeast, mouse, human, and *C. elegans *[11-16]. In human and *C. elegans*, LEM-2 interacts with lamins *in vitro *and requires lamins for its localization to the nuclear membrane [11,13]. Thus, LEM-2 is considered a member of the lamina network. LEM-2 is expressed in every human, mouse and *C. elegans *cell [11,13]. Its knockdown inhibits myoblast differentiation in mouse cells [16], and in *C. elegans *causes 15% embryonic lethality [13]. Lethality in *C. elegans *reaches 100% if the level of emerin is simultaneously reduced [13]. Emerin has been suggested to mediate transcriptional repression [17] by blocking access of transcription factors to genes [18]. LEM-2 is named for its LEM domain (LAP2, emerin, MAN-1), which interacts with the DNA-binding protein BAF-1 in human and *C. elegans*, illustrating one way that LEM-2 may interact with chromatin *in vivo *[13,19].

Our data show that the distal regions of the autosomes, which are called 'arms' despite the holocentric nature of *C. elegans *chromosomes, are associated with LEM-2 at the inner nuclear membrane, while the central regions are not. The large LEM-2 domains at the arms consist of smaller subdomains, which are characterized by a high density of repetitive sequences and a low density of genes. These subdomains are transcriptionally inactive, whereas the gaps between the subdomains are transcribed. Finally, we show that chromosome ends relocated to the center of a chromosome through an end-to-end chromosomal fusion remain associated with LEM-2, albeit at somewhat reduced levels. This shows that association with the nuclear membrane is characteristic of each chromosomal region, and only partly dependent on relative chromosome position. We provide a model of the spatial and functional arrangement of the *C. elegans *genome, which is physically supported by domain-scale and subdomain-scale association with the nuclear membrane.

## Results

### The integral membrane protein LEM-2 is localized to the nuclear membrane in every cell of *C. elegans *embryos

**Figure 1:**
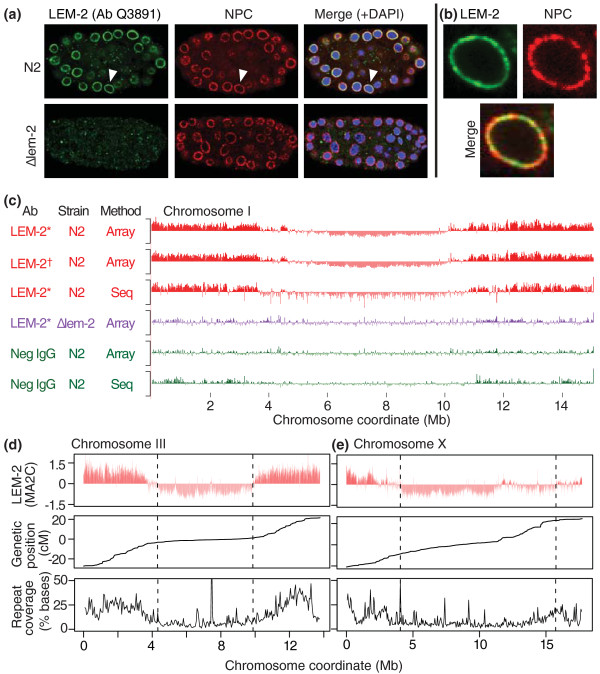
**Chromosome arms are associated with the nuclear membrane**. **(a) **Immunofluorescence analysis of *C. elegans *embryos with anti-LEM-2 antibodies (green), and the mAb414 antibody, which labels nuclear pore complexes (red). In the merged image, DNA stained by DAPI is shown in blue. The top row is wild-type N2 embryos; the bottom row is the *lem-2 *null mutant embryos. The arrowhead indicates the nucleus shown more closely in (b). **(b) **Enlarged image of the nucleus indicated by arrowhead in (a). **(c) **LEM-2 or negative control ChIP-chip (Array) or ChIP-seq (Seq) profiles. LEM-2* and LEM-2^† ^indicate antibody Q3891 and Q4051, respectively. Vertical bars in the tracks indicate average ChIP-chip signals (MA2C scores) or ChIP-seq signals (z-scores of (IP - input)) in 5-kb windows. The y-axis range is -2 to 2. **(d,e) **LEM-2 ChIP-chip signals (5-kb window MA2C scores), recombination rate (interpolated genetic position of genes in centimorgans (cM)), and coverage of repetitive sequences in 50-kb windows are shown on chromosomes III (d) and X (e). The other chromosomes are shown in Figure S2c in Additional file 1. Dashed lines indicate the edges of LEM-2 domains as judged by visual inspection.

We generated two rabbit polyclonal antibodies directed against the amino terminus of the *C. elegans *LEM-2 protein. The specificity of the antibodies was confirmed by western blotting, which detects a strong band at the expected size of 55 kDa in wild-type *C. elegans *embryos. The band was not present in extract prepared from *lem-2(ok1807) *null mutant animals (see Figure S1a in Additional file 1). By immunofluorescence microscopy, these antibodies exclusively stained the nuclear membrane of wild-type *C. elegans *embryos, whereas they did not produce specific signal in *lem-2 *mutant embryos (Figure 1a; Figure S1b in Additional file 1). Higher magnification of nuclei shows that LEM-2 apparently coats the entire nuclear membrane, with areas of slightly less signal at sites occupied by nuclear pore complexes (NPCs; Figure 1b; Figure S1c in Additional file 1). These results confirm the specificity of our antibodies and the nuclear membrane-specific localization of LEM-2 in *C. elegans *embryos. Therefore, in the sections below, we interpret association of genomic regions with LEM-2 to indicate that those regions are associated with the inner nuclear membrane.

### *C. elegans *autosome arms, but not central regions, are associated with the nuclear membrane

Using these validated anti-LEM-2 antibodies, we performed ChIP followed by tiling microarray analysis (ChIP-chip) or high-throughput sequencing (ChIP-seq) to identify regions associated with LEM-2 genome-wide. For ChIP, we used chromatin extracts from *C. elegans *mixed-stage embryos. Therefore, the ChIP signals we describe in the sections below represent the amalgamation of cell types that constitute the embryos. We normalized the ChIP-chip signals using MA2C [20], and ChIP-seq reads were converted to z-scores after accounting for the difference of genome coverage between LEM-2 ChIP and input control (Materials and methods). LEM-2 shows a striking association with the autosomal arms (Figure 1c). This pattern was reproduced in three biological replicates and is independent of the particular LEM-2 antibody used or the detection method employed (Figure 1c; Figure S2a in Additional file 1). In contrast, the negative-control ChIPs with non-specific antibody, or LEM-2 ChIP in the *lem-2 *null mutant embryos did not produce this pattern (Figure 1c). We confirmed that background signals seen in these control experiments are not related to LEM-2 signals (Figure S2b in Additional file 1). We observed strong LEM-2 association with the left and right arms of all five autosomes (Figure 1c,d; Figure S2c in Additional file 1). The LEM-2-associated regions, which we refer to as 'LEM-2 domains', typically extend inward approximately 4 Mb from both ends of the autosomes. In contrast, the central regions of the autosomes are almost completely devoid of LEM-2 association. These results demonstrate a common mode of LEM-2 association for *C. elegans *autosomes, in which the arm regions are attached to the nuclear membrane, and the central regions are likely looped out.

### Only the left end of the X chromosome is associated with the nuclear membrane

The X chromosome exhibits a pattern of LEM-2 interaction distinct from that of the autosomes. On X, only the left arm has a characteristic large LEM-2 domain, whereas the right arm has very weak LEM-2 associations (Figure 1e). Furthermore, the interaction strength of the left arm as represented by ChIP score is weaker than those of autosomes (Figure 1d,e; Figure S2c in Additional file 1). This suggests that the left arm is less frequently associated with LEM-2 than autosomal arms, or that the interaction is limited to a small proportion of cells in the embryos.

### The boundaries of regions associated with the nuclear membrane coincide with changes in repeat density and recombination frequency

The meiotic recombination rate and the density of repetitive sequences are known to differ between the chromosomal arms and central regions [21,22]. The meiotic recombination rate is high on arms and low in the central regions [21,23]. To directly determine the relationship between recombination and LEM-2 domains, we plotted genetic distance (centimorgans, cM) as a function of physical distance (Mb) across the chromosomes. Despite the fact that the LEM-2 ChIPs were performed in extracts prepared from embryos in which no cells are undergoing meiosis and nearly all cells are somatic, LEM-2 domains in autosomes correspond strongly to the regions with a high recombination rate. On the other hand, the central regions, which are mostly free of LEM-2 interaction, exhibit a low rate (Figure 1d; Figure S2c,d in Additional file 1). The relationship between meiotic recombination in germ cells and LEM-2 domains in somatic cells suggests that the nuclear organization of chromosomes may be similar in germ and somatic cells.

Repetitive sequences are over-represented on chromosomal arms in *C. elegans *[21,22]. Analysis of the proportion of annotated repetitive sequences in 50-kb windows showed that LEM-2 domains possess high densities of repetitive sequences (Figure 1d; Figure S2c,e in Additional file 1). The high LEM-2 levels observed at repeat-rich regions are not due to cross-hybridization associated with sequence redundancy because the association was also seen in ChIP-seq experiments in which we aligned only unique reads (Figure 1c).

The unique LEM-2 pattern on the X chromosome let us examine whether the high recombination rate and the high density of repeats are general characteristics of the LEM-2 domains. Repeats are concentrated on the left end of X, in the regions of high LEM-2 association, whereas the right end of X harbors fewer repetitive sequences and is only weakly associated with LEM-2 (Figure 1e). In contrast, we observed a difference between the autosomes and X with respect to recombination rate. The central region of the X has the highest recombination rate among all the chromosomes (Figure S2d in Additional file 1), but lacks LEM-2 association. Therefore, LEM-2 association and high meiotic recombination are separable characteristics at least on X, while high repeat density is a general characteristic of LEM-2 domains across the genome.

### The large domains associated with the nuclear membrane are punctuated by small gaps that are not associated with the membrane

**Figure 2:**
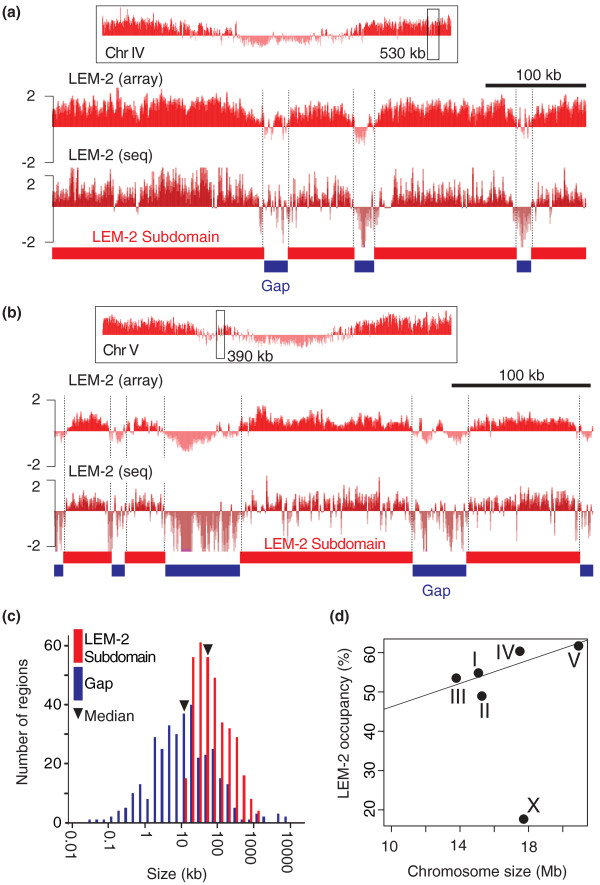
**Within large LEM-2 domains, a finer level of organization consists of LEM-2 subdomains and gaps**. **(a,b) **Representative LEM-2 subdomains on chromosomes IV (a) and V (b). Top panels with box indicate the chromosomal positions of regions shown below. Vertical bars in the tracks indicate ChIP-chip MA2C scores (-2 to 2) or ChIP-seq z-scores (-2 to 2). **(c) **Size distribution of LEM-2 subdomains and gaps. Subdomains or gaps were binned according to their size (log_10 _scale), and the number of regions for each bin are plotted. **(d) **Relationship between chromosome size and LEM-2 occupancy (total base pairs of LEM-2 subdomains divided by chromosome size (bp)). The line indicates a linear regression for autosomes by the least squares fit (intercept, 31.4; slope, 1.48).

The data presented above demonstrate the binding of LEM-2 to broad domains of chromosome arms. We next examined the pattern of LEM-2 binding within these domains more closely. We found that, within LEM-2 domains, there are many interruptions that result in generating smaller LEM-2-associated regions (Figure 2a,b). These regions, which we call 'LEM-2 subdomains', are typically greater than 10 kb in length, and exhibit continual LEM-2 binding. To rigorously define such LEM-2 subdomains, we converted ChIP scores to scores of either +1 or -1, and used a window-based method to identify domains with an average binary value over 0.8 for ChIP-chip or 0.4 for ChIP-seq (Materials and methods). Using a false discovery ratio <2.5%, we defined 360 LEM-2 subdomains (Table S1 in Additional file 2). These LEM-2 subdomains range in size from 11 kb to 1.3 Mb, with a median size of 58 kb (Figure 2c). Compared with subdomains, the regions between subdomains, which we call 'gaps', are generally smaller with a median size of 12 kb (Figure 2c; Table S2 in Additional file 1). Using this LEM-2 subdomain information, we assessed whether there is any quantitative difference in the proportion of each chromosome associated with LEM-2 (Figure 2d). We found that the longest chromosome (chromosome V) has the highest LEM-2 occupancy of approximately 60%, and that with the exception of the X chromosome, the general trend is that the occupancy correlates positively with chromosome size (*r *= 0.80, *P *= 0.11; Pearson's product-moment correlation).

LEM-2 subdomains exhibit characteristic distribution patterns across the chromosomes. First, larger subdomains are typically located closer to the chromosome ends and become smaller as a function of proximity to the centers (Figure S3a in Additional file 1). Second, gaps between subdomains are, in contrast, smaller when located close to the ends and larger when located close to the centers (Figure S3b in Additional file 1). Third, the average degree of LEM-2 association, as measured by ChIP scores, within subdomains gradually decreases with increasing proximity to the centers (Figure S3c in Additional file 1). Overall, the large LEM-2 domains consist of multiple subdomains, whose interaction with the nuclear membrane is stronger and more extensive near chromosome ends and becomes narrower, weaker and more sporadic closer to chromosome centers.

### Helitrons and satellite repeats are specifically associated with the nuclear membrane

**Figure 3:**
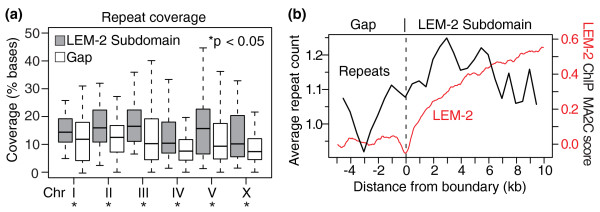
**Repeats are associated with the nuclear membrane**. **(a) **Coverage of repetitive sequences within LEM-2 subdomains or gaps. Percentages of bases covered by repetitive sequences are plotted. The bottom and top of boxes indicate the 25th and 75th percentiles, respectively, and bands in the boxes indicate medians. Whiskers indicate the lowest or the highest data points within 1.5 × interquartile range from the box. Wilcoxon rank sum test was used for the statistical analysis. **(b) **Average counts of repetitive sequences across LEM-2 subdomain-gap boundaries. The number of repeats were counted (according to each repeat's central coordinate) within sliding 1 kb windows (500 bp offset) for the 354 boundaries (Materials and methods). The average count in each window is plotted. Average LEM-2 ChIP-chip MA2C scores of sliding windows (100 bp window, 50 bp offsets) are also plotted.

If repetitive sequences are tightly associated with the nuclear membrane, the repeat density should be high in LEM-2 subdomains, but not in gaps. To focus on the subdomain-gap structure within the larger LEM-2 domains, we excluded the large central gaps from the analysis. Across all the chromosomes, LEM-2 subdomains exhibit higher levels of repeat coverage than gaps (*P *< 0.05, Wilcoxon test; Figure 3a). If a feature is associated with LEM-2 interactions, its occurrence should change at the boundaries between LEM-2 subdomains and gaps. We analyzed the average number of repeats in sliding windows across the boundaries. As expected, the average number of repetitive sequences increases across the boundaries, as the LEM-2 ChIP-chip score does (Figure 3b).

Although the difference of the repeat density between LEM-2 subdomains and gaps is significant (Figure 3a), its amplitude measured over all repeat families is relatively mild. To determine if some repeat families are more highly associated with the nuclear membrane than others, we analyzed repeat families individually. Of the various annotated repeats, satellite repeats and a class of rolling-circle transposons called helitrons [24] were much more enriched in LEM-2 subdomains relative to gaps (Figure S4a,b in Additional file 1; Discussion). In contrast, simple repeats, other classes of DNA transposons, low complexity repeats and retrotransposons (short interspersed elements (SINEs), long interspersed elements (LINEs) and long terminal repeats) show only a slight enrichment at LEM-2 subdomains (Figure S4c-h in Additional file 1).

### Genes tend to reside in gaps between LEM-2 subdomains

**Figure 4:**
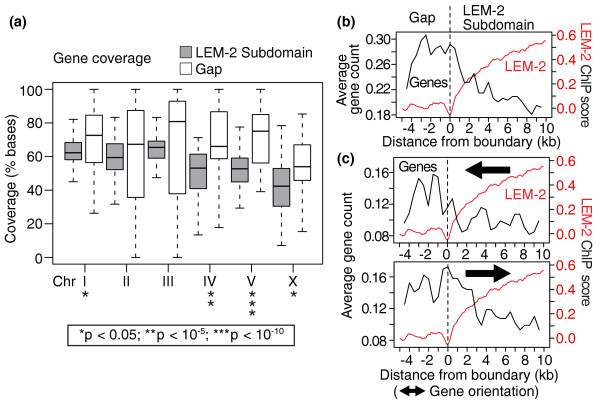
**Genes reside preferentially in the gaps between LEM-2 subdomains**. **(a) **Coverage of genes within subdomains or gaps. Percentages of bases covered by transcribed regions are plotted. Box plot representation and the statistical analysis are according to Figure 3a. **(b) **Average counts of coding genes across LEM-2 subdomain-gap boundaries. The number of translation start sites within sliding 1-kb windows (500-bp offset) were counted for the 354 boundaries. The average gene count in each window is plotted. Average LEM-2 ChIP-chip MA2C scores of sliding windows (100-bp window, 50-bp offsets) are also plotted. **(c) **Same as (b) but genes with the indicated orientations are plotted separately.

We tested whether gene density, which is highly variable across the *C. elegans *genome, differs between LEM-2 subdomains and gaps. Again, to focus on subdomain-gap structure within the larger LEM-2 domains, we excluded the central regions of the chromosomes from the analysis. We found that gene coverage is 12% higher in the gaps relative to the subdomains (median average of 68% in gaps versus 56% in subdomains; Figure 4a). Although the difference is not significant on chromosomes II and III, the rest of the chromosomes show clear enrichment of genes in gaps relative to LEM-2 subdomains (10^-11 ^<*P *< 0.05, Wilcoxon test). To confirm this association, we assessed the distribution of gene translation start sites across the LEM-2 subdomain-gap boundaries of all chromosomes. The profile confirmed that gene density decreases as one moves from gaps to subdomains and further revealed that translation start sites of genes preferentially occur just outside the LEM-2 subdomains (Figure 4b). A similar observation has been made at the boundary of lamin B1-associated domains in human cells. In human cells, there are more promoter regions oriented away from lamin B1-associated domains than orientated toward the domains [6]. Figure 4c shows that, unlike human, among genes that traverse LEM-2 subdomain boundaries, slightly more are oriented toward the LEM-2 subdomains than toward gaps in *C. elegans *(0.17 versus 0.12 genes per boundary, respectively), but the overall profiles are similar. Together, the data indicate that coding genes are over-represented in LEM-2 gaps, and that genes' translation start sites are preferentially located just outside of the LEM-2 subdomains regardless of their orientation.

### The genes in LEM-2 subdomains tend to be inactive, while those in gaps tend to be active

**Figure 5:**
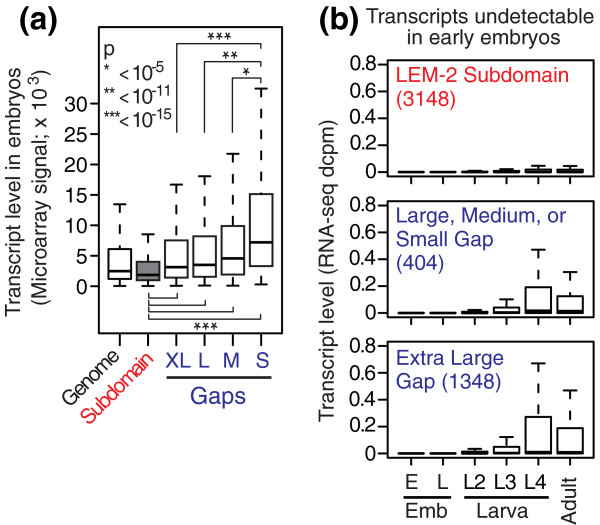
**Genes at the nuclear membrane are inactive**. **(a) **Expression level of genes within subdomains or gaps in mixed-stage embryos. Genes were categorized based on the size of gaps where they reside: extra large gap (XL), >1 Mb; large gap (L), 100 kb to 1 Mb; medium gap (M), 10 to 100 kb; and small gap (S), <10 kb. Box plot representation and the statistical analysis are according to Figure 3a. **(b) **Expression status during development for transcripts undetectable in early embryos. Transcripts were categorized in LEM-2 subdomains (top), large/medium/small gaps (middle) or extra large gaps (bottom) based on their start coordinates. We defined transcripts that were undetectable in early embryos as those with RNA-seq dcpm (depth of coverage per base per million reads) equals 0 in early embryos. E Emb, early embryo; L Emb, late embryo; L, larva stage; Adult, young adult.

We next asked if genes in LEM-2 subdomains and gaps are expressed. We measured transcript levels of *C. elegans *mixed-stage embryos in quadruplicate by microarrays and calculated the average level of expression among replicates for each transcript (Materials and methods). Next, we categorized transcripts as falling into LEM-2 subdomains (10,244 genes) or gaps (12,042 genes) based on the location of the corresponding gene's transcript start site (Table S3 in Additional file 2). The genes residing in gaps were further divided into four bins based on size of the gap in which they reside: extra large gaps (gap size >1 Mb; 9,016 transcripts), which correspond to the central regions of the chromosomes; large gaps (100 kb to 1 Mb; 1,612 transcripts); medium gaps (10 to 100 kb; 1,223 transcripts); and small gaps (<10 kb; 191 transcripts) (Table S3 in Additional file 2). The distribution of expression levels between LEM-2 subdomains and gaps (Figure 5a) revealed that genes associated with the nuclear membrane are poorly expressed relative to genes in gaps (*P *< 10^-15^, Wilcoxon test). These data demonstrate that genomic regions associated with LEM-2 are more likely to be inactive, whereas gaps are more likely to possess active genes.

### Silent genes at the nuclear membrane remain inactive during development

We examined whether the inactive state of genes at the nuclear membrane is stable during *C. elegans *development. We used publicly available RNA-seq data [25] to determine whether genes that are not expressed in early embryos become expressed in later developmental stages (Figure 5b). Embryonically silent transcripts in LEM-2 subdomains remain largely unexpressed in RNA-seq in later larval stages and young adults. In contrast, embryonically silent genes in gaps become expressed in later larval stages and young adults. These results suggest that most inactive genes at the nuclear membrane in embryos remain silent throughout development.

### The boundaries of LEM-2 subdomains generally match histone H3K27 trimethylation boundaries, but do not match H3K9 methylation patterns

**Figure 6:**
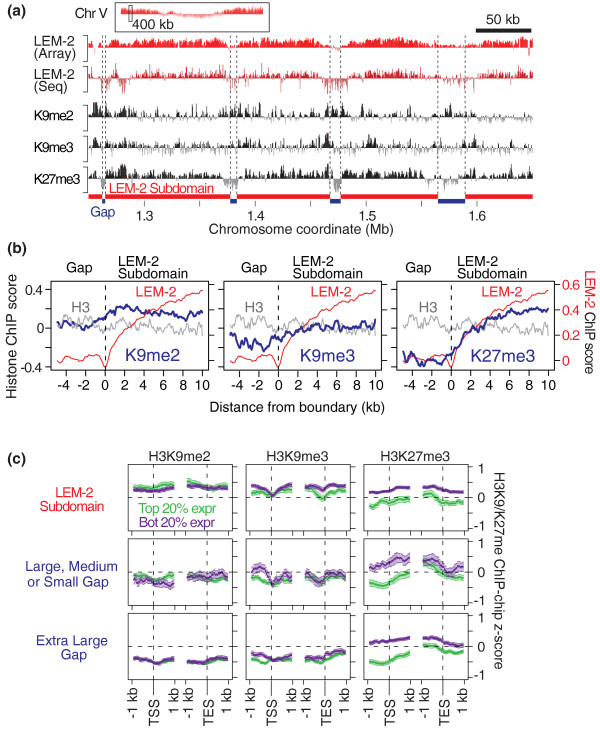
**H3K27me3 widely decorates LEM-2 subdomains except at active genes**. **(a) **A representative genomic region showing ChIP-chip signals for LEM-2, H3K9me2, H3K9me3 and H3K27me3. The top panel indicates the chromosomal position of the enlarged region. The y-axes represent MA2C scores (-2 to 2) for LEM-2 ChIP-chip or z-scores (-2 to 2) for LEM-2 ChIP-seq and histone modification ChIP-chip. **(b) **Average H3K9 and H3K27 methylation profiles at LEM-2 subdomain boundaries. Sliding window averages (100-bp window; 50-bp offset) of ChIP-chip z-scores for indicated histone modifications (blue) or control H3 (gray) are plotted. For comparison, sliding window averages of LEM-2 ChIP-chip MA2C scores (red) are also shown. **(c) **H3K9 and H3K27 methylation profiles of genes in LEM-2 subdomains or gaps. Top 20% highly expressed (Top 20% expr) or bottom 20% lowly expressed (Bot 20% expr) genes in mixed-stage embryos across the genome are separately plotted. Lines indicate sliding window averages (100-bp window; 50-bp offset) of ChIP-chip z-scores with vertical bars for 95% confidence intervals. TSS, transcript start site; TES, transcript end site.

H3K27 trimethylation (H3K27me3) is generally linked to transcriptionally inactive regions [26]. We therefore analyzed H3K27me3 status across the genome in early embryos (details about these histone modifications in *C. elegans *are described in our companion papers [27,28]). We found that H3K27me3 is enriched in LEM-2 subdomains but not in gaps (Figure 6a). Sliding window analysis across LEM-2 subdomain boundaries confirmed that H3K27me3 levels are generally higher in LEM-2 subdomains and the signal distribution mimics that of LEM-2 (Figure 6b). These results indicate that H3K27me3 largely decorates LEM-2 subdomains.

Other histone modifications linked to transcriptionally inactive regions are H3K9me2 and H3K9me3 [26]. In contrast to H3K27me3, we did not observe a clear relationship between the boundaries of LEM-2 subdomains and boundaries of H3K9me2 or H3K9me3 chromatin blocks (Figure 6a). Plotting average modification levels across LEM-2 subdomain boundaries confirmed that H3K9me2 and H3K9me3 levels are fairly flat across the boundaries, being slightly higher in subdomains than gaps (Figure 6b). Our data suggest that H3K9me2 and H3K9me3 do not correlate with LEM-2 association.

We then analyzed H3K27 methylation and H3K9 methylation distributions relative to the location and expression level of genes. While inactive genes in LEM-2 subdomains harbor high levels of H3K27me3, active genes within LEM-2 subdomains possess, like those in gaps, low levels of H3K27me3 (Figure 6c). In contrast, H3K9me2 and H3K9me3 levels are relatively elevated on genes in LEM-2 subdomains compared to genes in gaps, regardless of expression state of the gene. This suggests that genes at the nuclear membrane are more likely to harbor H3K9me2 and H3K9me3, but this is not explicitly linked to expression state. The difference of histone modification profiles between genes and LEM-2 subdomain boundaries could arise because the positions of genes are not finely aligned with the positions of LEM-2 subdomain boundaries (Figure 4b,c).

### RNA polymerase II, HTZ-1 and H3K4me3 occupy LEM-2 gaps

**Figure 7:**
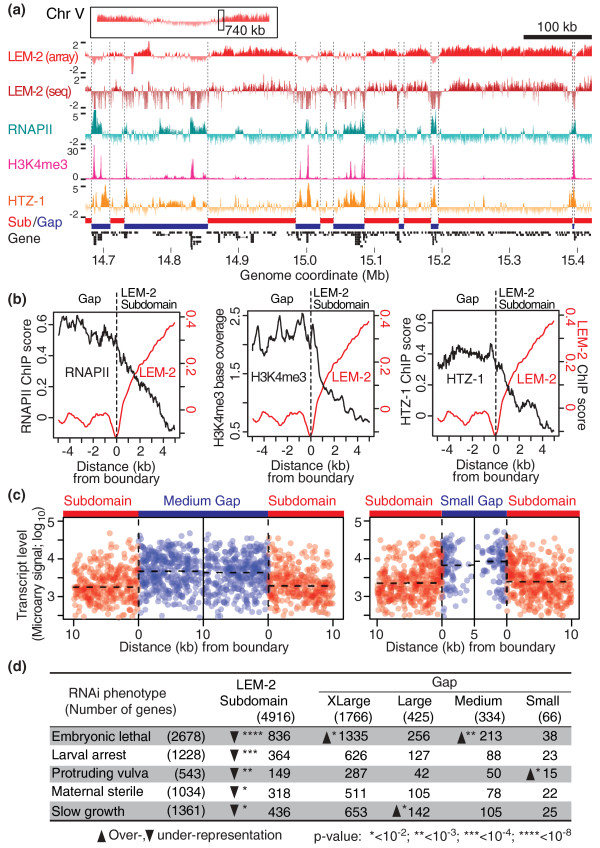
**Small gaps between LEM-2 subdomains are exceptionally transcriptionally active**. **(a) **A representative genomic region showing enrichment of RNA polymerase II (RNAPII), H3K4me3 and HTZ-1 in gaps between LEM-2 subdomains. The top panel indicates the chromosomal position of the enlarged region. RNAPII and HTZ-1 ChIP-chip data are shown as z-scores of log_2 _(ChIP/Input), whereas H3K4me3 ChIP-seq data are shown as normalized base counts. **(b) **Average RNAPII, H3K4me3, HTZ-1 and LEM-2 levels in subdomain-gap boundaries. For RNAPII and HTZ-1, sliding window averages (100-bp window; 50-bp offset) are shown, whereas for H3K4me3 the average of each base position is plotted. **(c) **Expression status of genes located near the boundaries. Each dot represents a transcript, whose abundance is shown on the y-axis and the distance from a boundary is shown on the x-axis. Boundaries between LEM-2 subdomains and medium gaps (left) or between subdomains and small gaps (right) are shown. The horizontal dashed bars indicate median transcript levels across 5-kb or 10-kb regions in gaps or subdomains. **(d) **RNA interference (RNAi) phenotypes of genes in LEM-2 subdomains and gaps. Numbers of genes with indicated RNAi phenotypes are shown. Chi-square test was used for statistical analysis with distribution of all genes with phenotypic annotations (shown in the header) as a background probability. Phenotypes annotated for more than 500 genes are listed.

To determine if the high RNA levels of genes in gaps (Figure 5a) reflect increased transcription, we examined the relationship between gaps and molecules that mediate transcription. We first compared the LEM-2-association profile with that of RNA polymerase II (RNAPII) [29]. The RNAPII level is generally low in LEM-2 subdomains, whereas gaps often include strong RNAPII binding (Figure 7a). Concordantly, the histone variant HTZ-1, which is often co-localized with RNAPII on the *C. elegans *genome [29], also has strong signals at the gaps. To further confirm the association between gaps and transcriptionally active status, we compared our data to the distribution of H3K4me3 (S Ercan, unpublished), which is generally associated with transcriptionally active genes [30]. H3K4me3 was strongly localized to gaps but rarely to LEM-2 subdomains (Figure 7a).

We further tested the relationship between markers of active transcription and gaps by plotting average levels of RNAPII, HTZ-1, and H3K4me3 across boundaries of nuclear membrane association (Figure 7b). The occupancy of each of these factors is high in gaps and sharply declines upon association of a chromosomal region with the nuclear membrane. Therefore, chromosomal regions that are likely looped out from the nuclear membrane are often bound by RNAPII, HTZ-1 and H3K4me3, whereas regions associated with the membrane rarely include these factors.

### Genes residing within very small LEM-2 gaps are expressed at exceptionally high levels

The variation in the sizes of LEM-2 gaps (Figure 2c) implies the existence of different-sized segments of DNA that likely loop out from the nuclear membrane. We explored whether the size of the loop might have any functional significance in relation to transcriptional activity. Strikingly, genes in the small gaps (those less than 10 kb) exhibit the highest range of expression levels, followed by genes in medium and then large gaps (Figure 5a).

To ask whether LEM-2 gaps are indeed looped out from the nuclear membrane, we examined the LEM-2 association status of three genes for which subnuclear localization in *C. elegans *embryos was determined by fluorescence *in situ *hybridization (FISH) [31] (Figure S5a in Additional file 1). The *baf-1 *gene, which was found mostly in the nuclear interior by FISH, is indeed located in chromosome III's central region, which lacks LEM-2 association (Figure S5b,c in Additional file 1). Strikingly, the *tbb-1 *gene, which was also found in the nuclear interior but closer to the nuclear periphery than *baf-1*, is located in a small LEM-2 gap (Figure S5b,d in Additional file 1). In contrast, the *pha-4 *gene, whose FISH signals were detected near the nuclear periphery in approximately 80% of the cases [31], is located in a LEM-2 subdomain (Figure S5b,e in Additional file 1). This analysis suggests that our LEM-2 ChIP results reflect positioning of chromosomal regions relative to the nuclear membrane. Finally, concordant with the observation that genes in small gaps are highly expressed, the small gap gene *tbb-1 *shows the highest expression among the three genes (Figure S5f in Additional file 1). These data support the idea that genes in small loops emerging from the nuclear membrane are highly transcribed.

A possible explanation for high expression in small gaps is that proximity to a boundary facilitates higher expression. We ruled this out, since higher transcription was not observed nearer to the boundaries of medium or small gaps (Figure 7c). Even in the 10-kb regions immediately adjacent to the boundary of membrane-associated chromatin, the median gene expression level in small gaps is significantly higher than the median in medium gaps (*P *< 10^-5^, Wilcoxon test). Therefore, some other property of small loops, perhaps a property inherent to the small loops themselves, supports higher levels of transcription.

### Genes essential for normal growth and viability are under-represented in LEM-2 subdomains and over-represented in gaps

We next explored if there is any bias for genes with critical developmental roles to reside at the nuclear membrane or in the gaps. We examined phenotypic annotations from previous RNA interference (RNAi) experiments (See Datasets in Materials and methods). We found that a set of RNAi phenotypes that characterize essential genes, such as 'embryonic lethal' and 'maternal sterile', are under-represented in LEM-2 subdomains (Figure 7d). In contrast, 'embryonic lethal' genes are over-represented in extra large and medium gaps, and 'slow growth' genes are over-represented in large gaps. Small gaps show over-representation of genes with a 'protruding vulva' phenotype, which is often associated with egg-laying defect [32]. A previous study reported that essential genes are more frequently found in chromosome centers in *C. elegans *[33], consistent with our finding 'embryonic lethal' genes enriched in extra large gaps. Our analysis revealed that this distribution is not simply correlated with position along chromosomes but with the membrane-association pattern, in which genes essential for normal growth and viability are distributed in gaps between the nuclear membrane-associated regions.

The genes linked to these RNAi phenotypes that occurred in LEM-2 gaps tend to be highly expressed. Among the top quartile of genes expressed in embryos were 81% of 'protruding vulva' genes in small gaps, 71% of 'embryonic lethal' genes in medium gaps, and 69% of 'slow growth' genes in large gaps. Thus, LEM-2 subdomain-gap structure is tightly linked to the expression of genes critical for animal development and function.

### Moving a chromosome arm to the center of a chromosome only slightly perturbs association with the nuclear membrane

**Figure 8:**
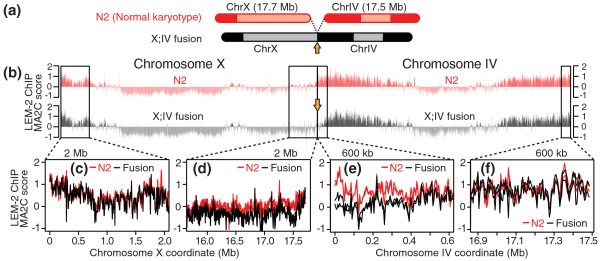
**Nuclear membrane association pattern of an X;IV fusion chromosome**. **(a) **Schematic representation of the wild-type (N2 strain) chromosomes X and IV, and the X;IV fusion chromosome *mnT12*. Large LEM-2 domains are indicated in dark colors. The arrow indicates the fusion point. **(b) **LEM-2 ChIP-chip signals on chromosomes X and IV in wild type (red, top) and on the X;IV fusion chromosome (black, bottom). Wild-type data are the average of four biological replicates. Data from the fusion strain are the average of two biological replicates. The arrow indicates the fusion point. The boxes indicate the regions shown more closely in (c-f). **(c-f) **LEM-2 ChIP-chip signal patterns in wild type (red) or the fusion chromosome strain (black) at the ends of chromosome X (c,d) or chromosome IV (e,f).

Our studies have shown that nuclear-membrane association selectively occurs at chromosome arms but also correlates with local characteristics of the genome. We therefore performed a test to determine if nuclear-membrane association is more dependent on a region's position along the chromosome or local signals. This test employed a strain possessing a fusion chromosome (*mnT12*), in which the right end of chromosome X is fused with the left end of chromosome IV [34] (Figure 8a). Homozygotes for the fusion chromosome were viable and fertile as reported [34], and we validated the strain by counting five bivalent chromosomes rather than the normal six in oocytes (Figure S6 in Additional file 1).

In the *mnT12 *strain, the right end of X and the left end of chromosome IV are now at the center of the new fusion chromosome (Figure 8a). If chromosomal location were key to determining membrane association, we would expect the new chromosomal center to be looped away from the nuclear membrane. Instead, we found that the regions of chromosomes X and IV associated with the nuclear membrane were almost identical to those observed in wild type (Figure 8b,c,f). Therefore, local features are the main determinants of localization to the nuclear membrane. This is further supported by the observation that the central region of chromosome IV, which in the fusion chromosome is now positioned quite far to the right in what would be considered the 'arm' on a normal chromosome, did not gain association with the nuclear membrane (Figure 8b).

However, we did observe a subtle difference in the fusion strain. The approximately 500-kb region of chromosome IV closest to the fusion point has a lower level of association with the membrane than it does in wild type (Figure 8e). This change was highly reproducible, and was confirmed in two independent biological replicates. A similar phenomenon was also observed on the X chromosome near the fusion point. Here, the dissociation extends approximately 1 Mb into the right arm of X (Figure 8d).

Because LEM-2 association decreased only within 1 Mb of the former chromosome ends, we wondered if a DNA- or chromatin-based determinant of LEM-2 interactions might be less prevalent near the ends than in the rest of the arm regions. We analyzed helitron and satellite repeat density in LEM-2 subdomains across the entire chromosome. Although highly enriched in LEM-2-associated chromatin, helitrons and satellite repeats are not particularly abundant in LEM-2 subdomains that are within 1 Mb of chromosome ends, even though these regions often show the highest level of LEM-2 association in wild-type strains (Figure S7 in Additional file 1). Indeed, this lower level of helitrons and satellite repeats is apparent on the left end of chromosome IV and the right end of X, where LEM-2 association decreased in the fusion chromosome. The data suggest that the chromosome ends are, at the sequence level, suboptimal for LEM-2 association and that the physical ends of chromosomes play a role in specifying membrane association. Overall, the results indicate that chromosomal association with the nuclear membrane is mostly dictated by local genomic or epigenomic characteristics, but may also require the physical ends of chromosomes for complete association with the nuclear membrane.

## Discussion

### Caveats of interpreting ChIP experiments

In this study, we used extract derived from a population of unsynchronized whole embryos to perform ChIP. Therefore, the pattern of nuclear membrane association we report may be an amalgamation of unique patterns of interaction that occurs in different cell types. It is likely that individual cells or cell types in *C. elegans *embryos have fewer membrane interactions or have patterns that differ in part from the pattern we report here, and it is further possible that the pattern in each cell type changes over the course of development. In addition, the interactions we report may occur only a relatively small fraction of the time, but be captured during the crosslinking treatment of the population of embryos. Finally, the interactions that we capture with LEM-2 almost certainly do not represent the only mode of interaction that chromosomes have with the nuclear membrane: ChIPs with other nuclear membrane components may yield different patterns. While future improvements in genome-wide methodology may allow us to overcome these caveats, the pattern we discovered is striking and correlates strongly with many biologically relevant chromatin marks, chromosomal activities and phenotypes. Our results yield several important new insights.

### LEM-2 interactions reveal a novel subdomain structure

The domain-subdomain structure of nuclear membrane association that we observe here has not been observed in mouse and human, despite the fact that similar genome-wide experiments were performed [6,7]. However, those studies used the DamID method to detect interactions, in which the resolution is approximately 1 kb, and the site of detection is limited to the Dam recognition sequence (GATC) [10]. Additionally, in DamID, smaller gaps can be adenine-methylated when the corresponding loops become close to the lamina at some points in a cell cycle, and therefore be detected as lamina-associated regions. It is possible that the ChIP-chip and ChIP-seq methods we used here provide a higher-resolution snapshot of physical association between LEM-2 and chromosomes and allow us to see the subdomains. Another possibility is that the difference between the pattern of LEM-2 association and lamin B1 or emerin association originates from a biological or functional difference between LEM-2, lamin B1 and emerin themselves. However, this is less likely since emerin, which shows a strikingly similar DamID profile with lamin B1 in human [6], is another nuclear inner-membrane protein that is functionally and genetically redundant with LEM-2 [13]. In any case, the domain-subdomain structure we observe here is a novel property of nuclear membrane-associated regions that may exist in other organisms, including mammals.

### A large portion of metazoan genomes is associated with the nuclear membrane

In *Saccharomyces cerevisiae*, the LEM-2-associated regions of the genome are limited mainly to the 10- to 20-kb subtelomeric regions, which comprise approximately 5-10% of each chromosome [15]. In contrast, LEM-2 domains in *C. elegans *typically extend approximately 4 Mb from the chromosome tips, which comprise about 25% (chromosome X) to 50% (autosomes) of each chromosome. This arm-specific pattern of nuclear membrane association was not observed in lamin B1-genome associations in *Drosophila melanogaster*, mouse, or human cells [6-8]. Therefore, the pattern of membrane association in *C. elegans *may be a consequence of the unique organization of its chromosomes. This organization, however, appears to have been conserved over at least 100 million years, since *Caenorhabditis briggsae *shares characteristics of chromosomal arms, including the high density of repetitive sequences and high rate of recombination [35]. It will be of interest to see if the nuclear membrane localization of the arms is also conserved.

In *Drosophila*, the precise coverage of lamina-associated regions is difficult to determine because low-resolution microarrays were used for mapping [8]. However, in human and mouse cells, lamin B1-associated regions cover, in total, approximately 40% of the genome [6,7]. This degree of membrane association is similar to the approximately 50% degree of association we observe in *C. elegans*. Although these proportions reflect membrane associations occurring in different cells, a general property of metazoan chromatin organization may be association of approximately half of the genome with the nuclear membrane.

### The X chromosome: a relationship between nuclear membrane association and dosage compensation?

**Figure 9:**
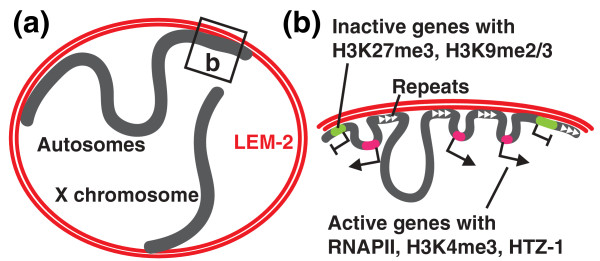
**Model for genome-nuclear membrane associations in *C. elegans***. **(a) **A model for large-scale chromosome arrangements mediated by the nuclear membrane. The large arm regions of autosomes are attached to the nuclear membrane, whereas the central portions of the chromosomes are looped out. For the X chromosome, the left arm is attached to the nuclear membrane, and the central and right portion of the chromosome are largely unattached. **(b) **A model for small-scale genome-nuclear membrane associations that underlie the large association domains. The small-scale associations often occur at regions with repetitive sequences and silent genes, and leave differentially sized gaps, which are likely looped out from the nuclear membrane. Among the loops, small loops are transcriptionally highly active.

In mammalian female cells, one of the two X chromosomes becomes inactivated, forming a 'Barr body' that frequently attaches to the nucleolus or the nuclear periphery [36]. In *C. elegans *hermaphrodites, each of the two X chromosomes undergoes chromosome-wide transcriptional repression of approximately two-fold to achieve dosage compensation [37,38]. Our study revealed that the *C. elegans *X chromosome lacks the degree of LEM-2 association typical of autosomes, suggesting a largely nucleoplasmic localization (Figure 9a). The left end of X that does associate with the nuclear membrane contains a region exhibiting autosomal characteristics, such as association with a H3K36 methyltransferase, MES-4 [39,40] and reduced binding of dosage compensation machinery [41]. Therefore, it seems unlikely that lamina-linked heterochromatinization functions to mediate dosage compensation in *C. elegans *as it does in mammals. However, *C. elegans *dosage compensation may be linked to the spatial arrangement of chromosomes through the association of X with membrane components other than LEM-2, or through the relative lack of membrane association for X. For example, our data demonstrate that small genomic regions that are likely to be looped out from the nuclear membrane support exceptionally high levels of transcription. Therefore, it is possible that the X chromosome's relative lack of interaction with the nuclear membrane contributes, through the lack of such transcriptionally active small loops, to the subtle down-regulation of X-linked genes in XX animals. This hypothesis can be tested by future experiments that examine interactions between the X and the membrane in males or animals defective in the dosage compensation process.

### Comparisons between ChIP and cytological observations

Previously, based on cytological observations in human cells, it had been hypothesized that large chromosomes tend to locate close to the nuclear periphery [42]. Although there is a positive trend between chromosome size and the LEM-2 occupancy (that is, proportion of LEM-2 binding region per chromosome) for the autosomes, the second-largest chromosome is the X, which had the lowest LEM-2 occupancy. Furthermore, the X;IV fusion chromosome, which is twice as large as any normal chromosome, did not acquire additional or longer LEM-2 interaction domains. Therefore, at least in *C. elegans *embryos, the peripheral versus internal position of chromosomes is not dependent on chromosome size. Our results do, however, support previous cytological observations linking chromosomes with low gene density and repetitive DNA, particularly satellite repeats, to positioning close to the periphery [43-46]. We extend this observation by identifying a recently identified class of transposons, helitrons [47], as families of repeats specifically associated with the nuclear membrane in *C. elegans*. While helitrons have not been identified in the human genome, they constitute >2% of the *C. elegans *genome [24,48]. A hallmark of helitrons is their ability to capture host genes [24]: therefore, during the evolution of the *C. elegans *genome, helitrons might influence distribution of genes within membrane-associated arm regions.

### *C. elegans *chromosome configuration is likely independent of Rabl orientation

Our data strongly suggest that in *C. elegans*, the arm regions of chromosomes are associated with nuclear membrane whereas the central regions are largely looped out (Figure 9a). A potentially related configuration, called Rabl orientation, has been observed cytologically in other organisms. In the Rabl orientation, centromeres and telomeres tend to be localized at opposite sides of interphase nuclei [49,50]. This orientation occurs as a result of the chromosome movements during anaphase, in which the centromeres lead the way into daughter cells and consequently localize toward the spindle pole, while the lagging telomeres localize distant from the pole [51]. However, the Rabl orientation is not likely to occur in *C. elegans*, since the chromosomes are holocentric and lack localized centromeres in the central regions. Instead, kinetochores form along the entire length of the chromosomes [52], making it unlikely that mitosis contributes to the pattern we observe.

### High transcriptional activity may be facilitated by small chromatin loops formed at the nuclear membrane

In chromosomal regions largely attached to the nuclear periphery, gaps in the association exhibit high transcription rates, and association with RNA polymerase II and active histone marks (Figure 9b). This is consistent in general with previous proposals that regions away from the nuclear periphery are transcriptionally active [1,5]. However, our data further demonstrated that small gaps, particularly those smaller than 10 kb, are much more active transcriptionally than larger gaps. Ten kilobases of nucleosomal DNA, if organized into a 30-nm chromatin fiber, is roughly 100 nm in length [53,54]. Since the diameter of nuclei in *C. elegans *embryos is about 2 μm, a 10-kb gap in chromatin would correspond to a position within 5% of the internal nuclear diameter from the periphery. This suggests that loci spatially close but not directly attached to the nuclear membrane are more transcriptionally competent than those that extend deeply into the nucleoplasm. It is possible that active transcriptional machineries may be concentrated in a nucleoplasmic space just underneath the nuclear membrane. Alternatively, transcription occurring at the membrane may facilitate, through a conformational change of chromatin, dissociation of DNA regions from the nuclear membrane. Another possibility is that small loops anchored by nuclear membrane interaction allow local recycling of transcriptional components, leading to higher transcription frequency. Such recycling has been observed in other systems [55], but has not yet been linked to membrane proximity.

## Conclusions

By probing interactions between the genome and the inner nuclear membrane protein LEM-2, we propose a general model for the arrangement of chromosomes in *C. elegans *interphase nuclei. The autosomal arm regions, which span 4 to 5 Mb on each chromosome end, are attached to the nuclear membrane, whereas the central regions, also megabases in length, are likely looped out (Figure 9a). The large, membrane-associated domains consist of multiple subdomains that are punctuated by gaps (Figure 9b). These gaps are genomic regions of various size detached from the nuclear membrane, within the context of the membrane-localized arm regions. Small gaps possess highly expressed genes, suggesting that small regions looped out from the nuclear membrane are especially amenable to highly efficient transcription. We found that association with the nuclear membrane is determined mostly by local signals, but can be influenced by chromosomal position.

## Materials and methods

### Antibodies, strains and worm growth

Affinity purified rabbit polyclonal antibodies (Q3891 and Q4051) were produced against amino acids 1 to 100 of LEM-2 by genetic immunization at SDIX (catalogue number 4854.00.02, Newark, Delaware, USA). Mouse monoclonal antibodies for H3K9me2 (clone 6D11), H3K9me3 (clone 2F3), and H3K27me3 (clone 1E7) are kindly provided by Dr. Hiroshi Kimura (Osaka University, Japan). Anti-H3K4me3 antibody (mouse monoclonal, 305-34819, Wako Chemicals USA, Richmond, VA, USA), anti-nuclear pore complex antibody (mouse monoclonal, mAb414, ab24609, Abcam, Cambridge, MA, USA), anti-histone H3 antibody (rabbit polyclonal, ab1791, Abcam) and a rabbit polyclonal negative control antibody (ab46540, Abcam) were commercially available.

N2 (wild type), *lem-2 *mutant (*ok1807 *allele, VC1317 strain), and IV;X fusion (*mnT12 *allele, SP646 strain) worms were obtained from the Caenorhabditis Genetics Center (University of Minnesota, USA). Standard worm growth techniques were used to obtain embryos from worms grown in S liquid media [56].

### Microscopy

For immunofluorescence, *C. elegans *embryos collected from gravid worms by hypochlorite treatment were frozen in liquid nitrogen and stored in methanol at -20°C until use. Embryos were rehydrated in PBST (phosphate-buffered saline, 0.1% Tween 20) for 15 minutes followed by fixation with 1% formaldehyde. Fixed embryos were blocked by goat sera and then incubated with PBST containing rabbit anti-LEM-2 (1:1,000 dilution) and mouse mAb414 antibodies (1:400 dilution). Immunocomplexes were fluorescently labeled using secondary antibodies conjugated with Alexa 488 (anti-rabbit) or Texas Red (anti-mouse), and DNA was stained with 4',6-diamidino-2-phenylindole (DAPI). Fluorescent signals were captured using a TCS SP2 laser scanning confocal microscope (Leica Microsystems, Bannockburn, IL, USA).

To visualize chromosomes in oocytes, adult worms were fixed by 4% formaldehyde and then incubated with DAPI for 10 minutes. Signals were detected as described above.

### Western blotting

Proteins from embryos were separated by an SDS-polyacrylamide gel and transferred to a polyvinylidene fluoride (PVDF) membrane. Blocked membranes were incubated with anti-LEM-2 antibodies (1:5,000 dilution) and then with secondary antibody conjugated with horseradish peroxidase (1:10,000 dilution). Signals were detected by ECL Plus Western Blotting Detection system (GE Healthcare, Piscataway, NJ, USA) through autoradiography. Total proteins on membranes were stained by Coomassie Brilliant Blue dye for loading controls.

### ChIP-chip

The ChIP-chip experiments performed in this study are summarized in Table S4 in Additional file 2. Histone H3 and methyl mark ChIP-chip experiments done with early embryo extracts were performed as previously described [40]. LEM-2 or negative control ChIP in mixed-stage embryos were performed as previously described [41] except for the following modifications: anti-LEM-2 antibodies or a control antibody (10 μg) were immobilized onto Protein A-conjugated sepharose beads, which were subsequently incubated at 4°C for over 12 hours with chromatin extract corresponding to 0.5 mg of total proteins.

Amplification of DNA from histone H3 or methyl mark ChIP was performed using ligation-mediated PCR (LM-PCR) as described previously [40]. LM-PCR for LEM-2 or control ChIP experiments were performed as described below. First, DNA from ChIP or input was blunt-ended by End Repair Enzyme mix (ER0720, Epicentre, Madison, WI, USA) in the presence of dNTP. To add deoxyadenosine at the 3' ends, DNA was treated with exonuclease-(-) Klenow fragment in the presence of dATP. DNA fragments were ligated with deoxythymidine-overhang linkers and then amplified by PCR with the longer linker oligonucleotide as a primer (Table S5 in Additional file 2).

For tiling microarray analysis of LEM-2 and control ChIP, the amplified ChIP or input DNA was labeled with Cy3 or Cy5 by reaction with Cy3- or Cy5-conjugated random primers in the presence of dNTPs and exonuclease-(-) Klenow fragment. Dye orientation of experiments is described in Table S4 in Additional file 2. The labeled DNA was hybridized to a *C. elegans *tiling array (see below) at 42°C for 16 to 20 hours. Microarrays were scanned by using a GenePix 4000B Scanner with associated software (Molecular Devices, Sunnyvale, CA, USA). Raw signal intensities of the images were extracted by using NimbleScan software v2.5 according to the NimbleScan User's Guide (Roche NimbleGen Inc., Madison, WI, USA). Complete procedures employed for the microarray hybridization and signal detection are described in the NimbleGen Arrays User's Guide (ChIP-chip Analysis, version 3.1, 27 May 2008 [57]). For hybridization of histone H3 or methyl mark ChIP experiments, essentially the same procedures were employed, but at Roche NimbleGen Inc. as previously described [40].

### ChIP-seq

The ChIP-seq experiments performed in this study are summarized in Table S4 in Additional file 2. Preparation of LEM-2 or control IgG ChIP DNA was described in the 'ChIP-chip' section. For H3K4me3 ChIP (S Ercan, unpublished), 3 μg of antibodies were incubated with the embryo extract corresponding to 1 mg of total protein at 4°C, and the immunocomplex was isolated by anti-mouse IgG-immobolized Dynabeads (M280, Invitrogen, Carlsbad, CA, USA). ChIP DNA from the immunocomplex was purified as previously described [41].

For sequencing library preparation, the LEM-2 ChIP DNA or input DNA was blunt-ended as described in the 'ChIP-chip' section. For H3K4me3 ChIP DNA, fragments were blunt-ended by a custom mixture of polynucleotide kinase, Klenow fragment, and T4 DNA polymerase. All ChIP or input DNA fragments were then modified to provide an A-overhang as described in the 'ChIP-chip' section, ligated with the single-end adaptor (Illumina, San Diego, CA, USA), and then amplified by PCR with single-end PCR primers (Table S5 in Additional file 2). Amplicons were loaded to a 2% agarose gel, and DNA between 300 and 500 bp (LEM-2, control IgG and input DNA) or 200 and 400 bp (H3K4me3) was recovered from the gel. DNA samples were sequenced by Genome Analyzer II (Illumina) according to the manufacture's protocol at the High Throughput Sequencing Facility at the University of North Carolina at Chapel Hill. Raw sequenced reads that fulfilled the Illumina's default quality control were processed as described in the 'Processing of ChIP-seq data' section.

### Tiling array design and processing of ChIP-chip data

The *C. elegans *tiling array used in this study (080922_modEncode_CE_chip_HX1, Gene Expression Omnibus (GEO) [58] accession ID [GEO:GPL8647]) was designed based on WS170 (ce4) genome assembly and contains 1.9 million 50-mer probes with median spacing of 50 bp (center-to-center) across the genome.

For the histone H3 and methyl mark ChIP-chip data, average z-scores of [ChIP signal]/[Input signal] between replicates were calculated as previously described [40] and used in the sliding window analysis. For LEM-2 and control ChIP-chip data, we employed the MA2C program [20]. MA2C normalized the log_2 _ratio (log_2_([ChIP signal]/[Input signal])) of each probe based on the probe behavior estimated by its GC content, and then smoothed the value by assigning the median across sliding windows of 300 bp. The resultant values are MA2C scores. For data analysis, we combined four replicates of LEM-2 ChIP-chip datasets or two replicates of control IgG datasets (Table S4 in Additional file 2). For this purpose, we used MA2C, which returned the median MA2C score of a pool of normalized log_2 _ratios from all the replicates in each 300-bp sliding window [20]. The combined MA2C scores were used for subsequent LEM-2 subdomain calling and sliding window analyses. For visualization in figures, either combined or single experiment MA2C scores were used as indicated in the figure legends. To facilitate chromosome-scale data visualization by reducing the number of data points, MA2C scores were averaged within non-overlapping 5-kb windows across the genome.

### Processing of ChIP-seq data

Using the MAQ program [59], we aligned only unique reads to the *C. elegans *reference genome (ce4, WS170), disallowing mismatches. According to the size of DNA excised from the gels for the sequencing, we extended the aligned reads computationally to 200 bp (H3Kme3) or 300 bp (LEM-2, control IgG and input DNA). The numbers of the aligned reads and the coverage of the extended reads on the genome were: LEM-2, 6.0 million (18% coverage); control IgG, 2.5 million (coverage 7.6%); H3K4me3 replicate 1, 11.6 million (23% coverage), H3K4me3 replicate 2, 4.6 million (9.2% coverage); and input DNA, 10.0 million (30% coverage). Since the two replicates of the H3K4me3 datasets were highly concordant with each other, we used only replicate 1 for subsequent analyses.

To obtain the 'base-count' profile for LEM-2 ChIP, control IgG ChIP and H3K4me3 ChIP, the number of the extended reads overlapping each base of the genome was counted. For input DNA, we randomly selected a number of aligned reads corresponding to the number of those in LEM-2 or control IgG ChIP-seq datasets, and then generated base-count profiles for normalization as described below.

To normalize the LEM-2 and control IgG datasets using the input DNA data, we subtracted base-count of the input DNA from that of LEM-2 or control IgG ChIP at each base. The subtracted base-counts were transformed to z-score using the mean and the standard deviation (SD; z = (Base-count - Mean)/SD). Finally, z-scores were smoothed by averaging values in a 300-bp sliding window with a 50-bp offset. The normalized z-score at every 50 bp was used for visualization in figures and for subsequent LEM-2 subdomain calling.

To normalize H3K4me3 ChIP-seq data, each base-count was divided by the average base count across the genome, and then plotted for visualization.

### LEM-2 subdomain calling

To define LEM-2 subdomains, we binarized the replicate-combined LEM-2 ChIP-chip data by transforming positive or negative MA2C scores to +1 or -1, respectively. We averaged the binarized values in 200-probe (approximately 10 kb) windows, sliding one probe (50-bp offset) across the genome to subsequently identify windows with high average binary values. In this analysis, we used any genomic probe including those overlapping with repetitive sequences to generate windows across the genome. We then performed the same procedure for the replicate-combined control IgG ChIP-chip data to estimate the number of false-positive windows. We defined LEM-2 positive windows when a window binary value is over 0.8, at which the ratio of the number of positive control IgG windows to that of positive LEM-2 windows (false discovery ratio) is <2.5% (Figure S8 in Additional file 1). We then joined any overlapping (≥1 bp) windows to generate ChIP-chip-derived LEM-2-positive regions.

We performed essentially the same procedure to generate ChIP-seq-derived LEM-2-positive regions. We first binarized the z-scores of the LEM-2 or control IgG ChIP-seq datasets. Using a window size of 10 kb with an offset of 50 bp, which is comparable to the ChIP-chip window setting, we obtained a window average. We defined ChIP-seq-derived positive windows with a threshold value over 0.4, which fulfills false discovery ratio <2.4% (Figure S8 in Additional file 1), and then joined overlapping (≥1 bp) windows.

Finally, we excluded platform-specific false positive windows, such as those solely derived from the ChIP-chip probes overlapping with repetitive sequences, by discarding non-overlapping positive windows between ChIP-chip and ChIP-seq. We then combined ChIP-chip- and ChIP-seq-derived positive regions if they overlapped (≥1 bp) and generated 360 regions, which we defined as 'LEM-2 subdomains'. Chromosome coordinates for these LEM-2 subdomains are listed in the Table S1 in Additional file 2.

### Sliding window analysis

The 354 boundaries we used for the sliding window analyses are listed in Table S6 in Additional file 2. The 354 boundaries correspond to the left edges of all LEM-2 subdomains (360), except 6 subdomains located at the very left end of each chromosome. To avoid analyzing same data points twice, we did not merge left and right edges. Both sides of boundaries provide essentially the same result. When gaps are less than 5 kb, we limited the analyses to the size of gaps for the boundaries. All subdomains are greater than 10 kb. For the analysis of repetitive sequences across the boundaries (Figure 3b), all annotated repetitive sequences (99,113 total repeats) were analyzed. For the analysis of translation start sites (Figure 4b, c), we used only non-redundant coding sequences (22,355 out of 30,497) by accepting only one coding sequence when more than one transcript share the same start and end sites. The source of the annotation is described in 'Datasets' below. For sliding window analyses of ChIP data (Figure 6b, 7b), we used replicate-combined ChIP-chip MA2C scores for LEM-2 analysis; replicate-combined ChIP-chip z-scores for H3K9me2, H3K9me3, H3K27me3, HTZ-1 and RNAPII (see 'Datasets'); and ChIP-seq base-count profile for H3K4me3 replicate 1. Only unique probes in ChIP-chip data were analyzed.

For sliding window analyses around transcript start and end sites (Figure 6c), genes that are ranked in the top or bottom 20% expression levels among all genes analyzed by microarray were chosen. To avoid analyzing same data points twice in these plots, we removed genes less than 2 kb in length or genes overlapping with other genes between the transcript start site minus 1 kb and transcription end site plus 1 kb. The numbers of genes analyzed are (top 20%/bottom 20%): 141/293 for LEM-2 subdomains; 96/27 for small, medium and large gaps; and 215/97 for extra large gap.

### Expression profiling

*C. elegans *embryos were suspended in Trizol reagent (Invitrogen) with chloroform. RNA was isolated from the aqueous phase and purified by isopropanol precipitation. We obtained RNA from four biological replicates (Table S4 in Additional file 2). Subsequent processes, including cDNA synthesis, microarray hybridization, signal detection and signal normalization, were performed at Roche NimbleGen, Inc. Briefly, cDNA was synthesized from each biological replicate with oligo d(T) primer and hybridized individually to a microarray containing 72,000 probes (60-mer) corresponding to 23,336 genes (3 probes per gene). The four samples were hybridized to four individual arrays on a single slide (080128_worm170_modENCODE_expr, [GEO:GPL8673] [58]). Signals from each biological replicate were processed using quantile normalization method [60] and assigned to each gene using the Robust Multichip Average (RMA) algorithm [61,62] through NimbleScan software. The averaged RMA values across the four replicates were used for analyses.

### Datasets

All ChIP-chip, ChIP-seq and expression microarray experiments performed in this study are listed in Table S4 in Additional file 2. These data are publically available at the GEO website [58] [GEO:GSE25933], and at the modENCODE Data Coordinating Center (DCC) website [63] under individual accession IDs listed in Table S4 in Additional file 2. Interpolated recombination rate and RNAi phenotypes assigned to each gene in the genome (ce4 assembly) were obtained from WormMart [64]. Genomic coordinates and classes of repetitive sequences defined by RepeatMasker [65] and coding genes defined by WormBase [66] were obtained from the UCSC genome browser (ce4 assembly) [67]. RNA-seq data sets generated by Drs. LaDeana Hillier and Robert Waterston [25] were obtained from the modENCODE DCC website [63]. Dataset IDs for these RNA-seq experiments are: modENCODE_2473 (early embryo); modENCODE_2475 (late embryo); modENCODE_2466 (L2); modENCODE_2467 (L3); modENCODE_2468 (L4); modENCODE_2470 (young adult). Datasets for RNAPII and HTZ-1 ChIP-chip experiments [29] were available under [GEO:GSE10201] [58]. Since the RNAPII and HTZ-1 ChIP-chip experiments were performed using microarrays designed for the ce2 (WS120) assembly [29], we converted the genomic coordinates to those of the ce4 assembly.

## Abbreviations

Bp: base pair; ChIP: chromatin immunoprecipitation; ChIP-chip: ChIP followed by tiling microarray analysis; ChIP-seq: ChIP followed by high-throughput sequencing; cM: centimorgan; DamID: DNA adenine methyltransferase identification; DAPI: 4',6-diamidino-2-phenylindole; DCC: Data Coordinating Center; FISH: fluorescence *in situ *hybridization; GEO: Gene Expression Omnibus; H3K4/K9/K27me2 or me3: H3K4/K9/K27 di, or trimethylation; LEM: LAP2, Emerin, and MAN-1; LINE: long interspersed element; RNAi: RNA interference; RNAPII: RNA polymerase II; SINE: short interspersed element.

## Competing interests

The authors declare that they have no competing interests.

## Authors' contributions

KI performed experiments except histone modification ChIP and analyzed the data. TAE carried out ChIP-chip of H3K9 and H3K27 methylation. SS helped to draft the manuscript. KI and JDL designed experiments and wrote the manuscript. All authors read and approved the final manuscript.

## Supplementary Material

Additional file 1 **Figures S1 to S8**. Figure S1: antibody characterization. Figure S2: LEM-2 domains on individual chromosomes. Figure S3: distribution of LEM-2 subdomains along chromosomes. Figure S4: coverage of different types of repetitive sequences in LEM-2 subdomains and gaps. Figure S5: LEM-2 association status of genes for which subnuclear positions were previously determined by fluorescent *in situ *hybridization (FISH). Figure S6: validation of a strain with a fusion chromosome. Figure S7: coverage of helitrons and satellite repeats in LEM-2 subdomains along chromosomes. Figure S8: LEM-2 subdomain calling. Click here for file

Additional file 2 **Tables S1 to S7**. Tables are in individual tabs in Additional file 2 (Microsoft Excel file). Table S1: LEM-2 subdomains. Table S2: gaps between LEM-2 subdomains. Table S3: the number of genes in LEM-2 subdomains and gaps. Table S4: ChIP-chip, ChIP-seq and expression profiling performed in this study. Table S5: oligonucleotide sequences used in this study. Table S6: LEM-2 subdomain-gap boundaries analyzed in this study. Table S7: genome coordinates for chromosome arms and central regions. Click here for file
