# Supplementary material for: Caenorhabditis elegans chromosome arms are anchored to the nuclear membrane via discontinuous association with LEM-2
Source: Genome Biol. 2010 Dec 23;11(12):R120. doi: 10.1186/gb-2010-11-12-r120 (PMC3046480; doi:10.1186/gb-2010-11-12-r120)
Supplement: Additional file 1 — Figures S1 to S8. Figure S1: antibody characterization. Figure S2: LEM-2 domains on individual chromosomes. Figure S3: distribution of LEM-2 subdomains along chromosomes. Figure S4: coverage of different types of repetitive sequences in LEM-2 subdomains and gaps. Figure S5: LEM-2 association status of genes for which subnuclear positions were previously determined by fluorescent in situ hybridization (FISH). Figure S6: validation of a strain with a fusion chromosome. Figure S7: coverage of helitrons and satellite repeats in LEM-2 subdomains along chromosomes. Figure S8: LEM-2 subdomain calling. [file gb-2010-11-12-r120-S1.pdf]

Figure S1

Ikegami et al.

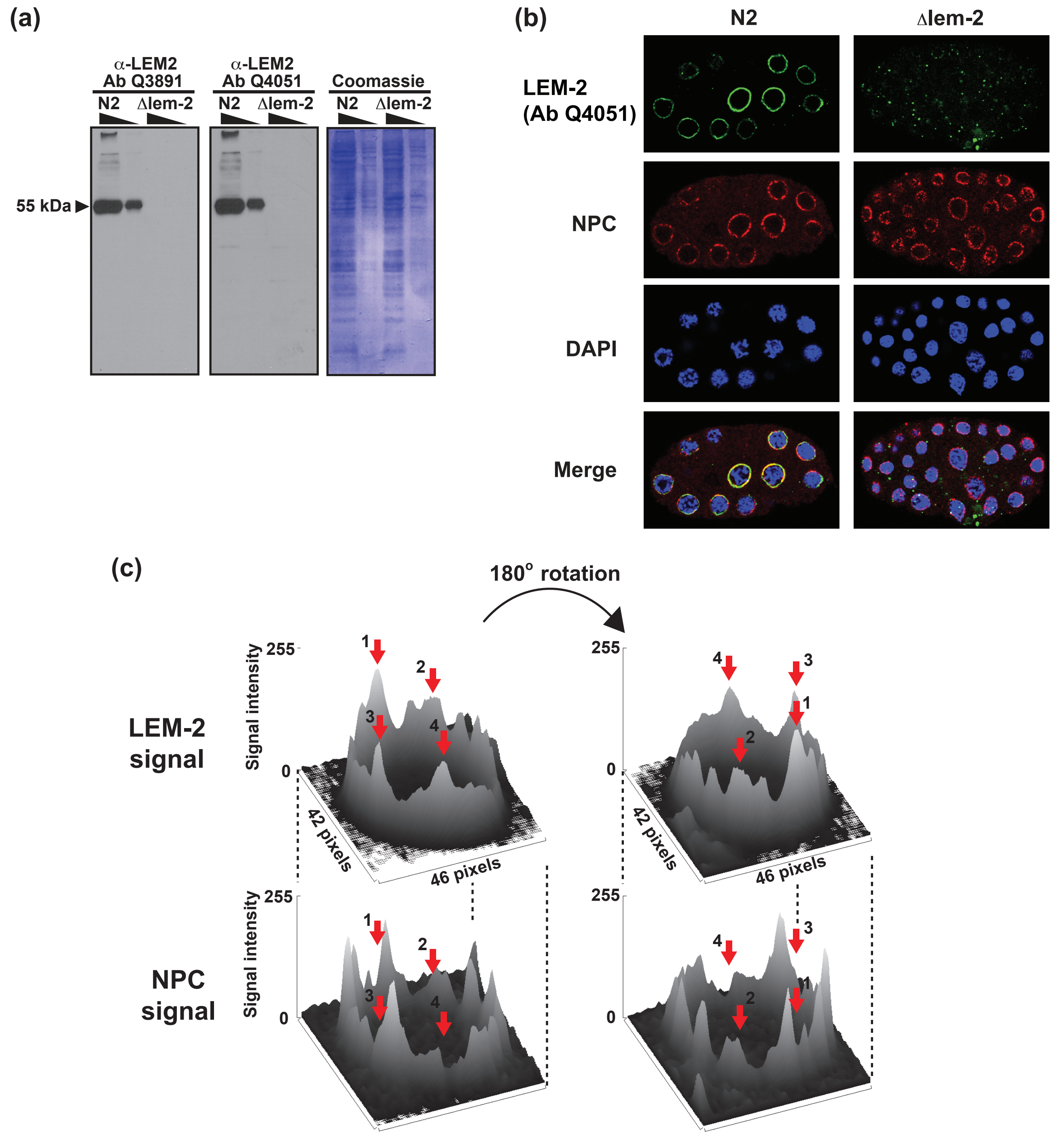

Figure S1. Antibody characterization

(a) Western blot analysis with anti-LEM-2 antibodies showing their specificity to LEM-2. Proteins from the wild-type (N2) or the *lem-2* null mutant embryos were analyzed at two different dilutions. The blot was also stained by Coomassie dye to show equal loading. The arrowhead indicates LEM-2. **(b)** Immunofluorescence analysis of *C. elegans* embryos stained for LEM-2 by anti-LEM-2 antibody (Q4051), nuclear pore complexes (NPCs) by mAb414 antibody and DNA by DAPI. The left column is the wild-type N2 embryos; the right column is *lem-2* null mutant embryos. Staining with the other anti-LEM-2 antibody (Q3891) is shown in Figure 1a. **(c)** Quantification of immunofluorescence signals shown in Figure 1b. Analysis was performed using Image J software (www.rsbweb.nih.gov/ij/). Arrows indicate representative LEM-2 signal peaks in the top panel (LEM-2) and the corresponding position in the bottom panel (NPC).

Figure S2 Ikegami et al.

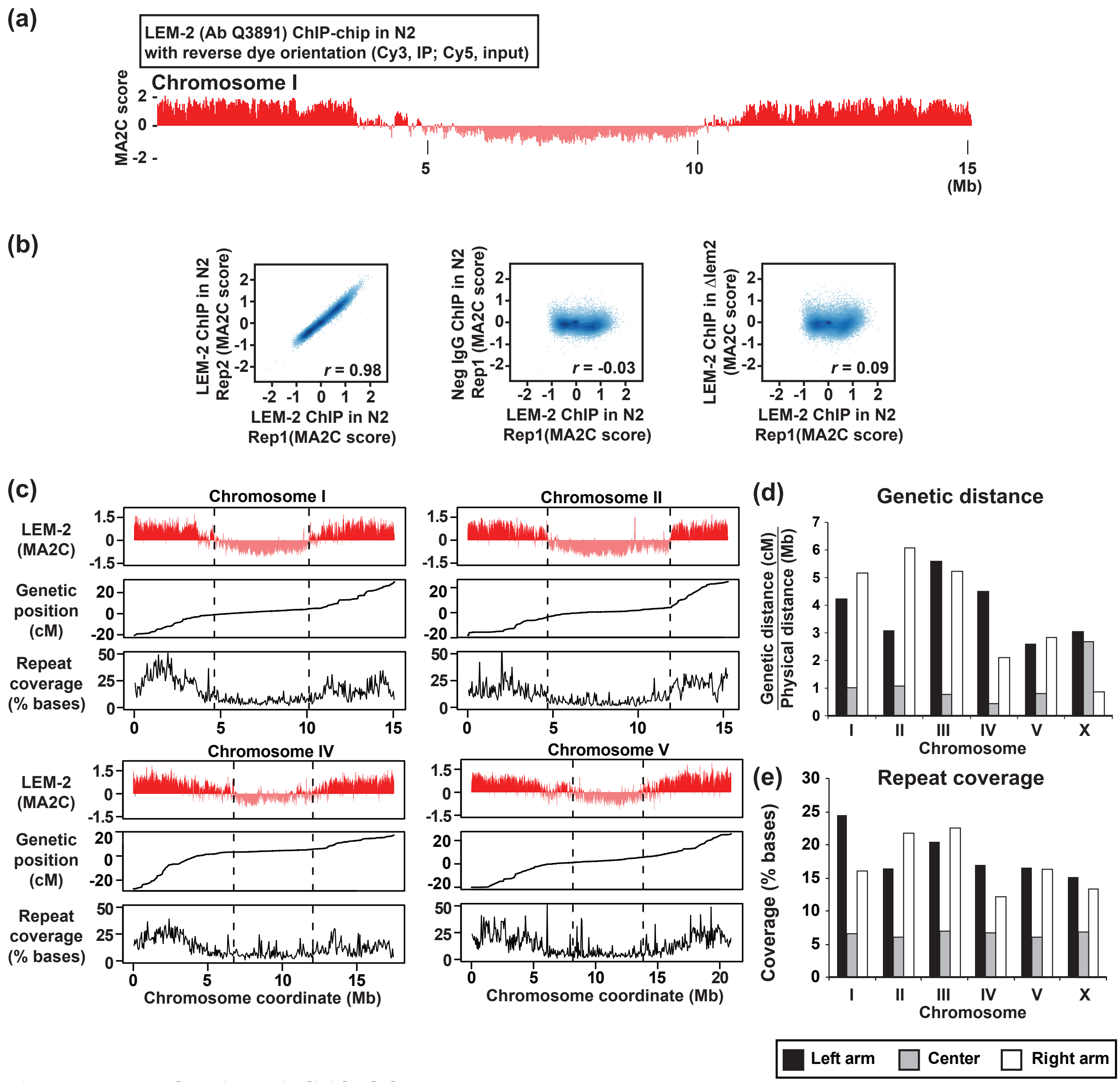

Figure S2. LEM-2 domains on individual chromosomes

(a) ChIP-chip signal (MA2C scores) on chromosome I obtained by co-hybridizing Cy3-labeled LEM-2 ChIP DNA and Cy5-labeled input DNA on a microarray. This dye orientation is the reverse of conventional ChIP-chip (Figure 1c), but reproduced the arm-rich LEM-2 signal pattern. **(b)** Relationship between replicates of LEM-2 ChIP-chip (left), between LEM-2 ChIP-chip and negative control IgG ChIP (center), and between LEM-2 ChIP-chip in N2 and in *lem-2* null mutant (right). Average MA2C scores in 5 kb windows are plotted. *r*, correlation coefficient. **(c)** Comparison of LEM-2 signal, the recombination rate, and the abundance of repetitive sequences on chromosomes I, II, IV and V. Chromosome III and X are shown in Figure 1d and e. 1st track: average LEM-2 ChIP-chip MA2C score within 5 kb windows. 2nd track: recombination rate as presented by the interpolated genetic position (centimorgan (cM)) of genes as a function of physical position. 3rd track: the coverage of repetitive sequences within 50-kb windows. Dashed lines indicate the edges of LEM-2 domains as judged by visual inspection. These edges were used to define arms and central regions in (d) and (e), and their genome coordinates are listed in Table S7. **(d)** Genetic distance between left-edges and right-edges of arm or central regions normalized by physical distance. **(e)** Coverage of repetitive sequences in arm or central regions.

Figure S3 Ikegami et al.

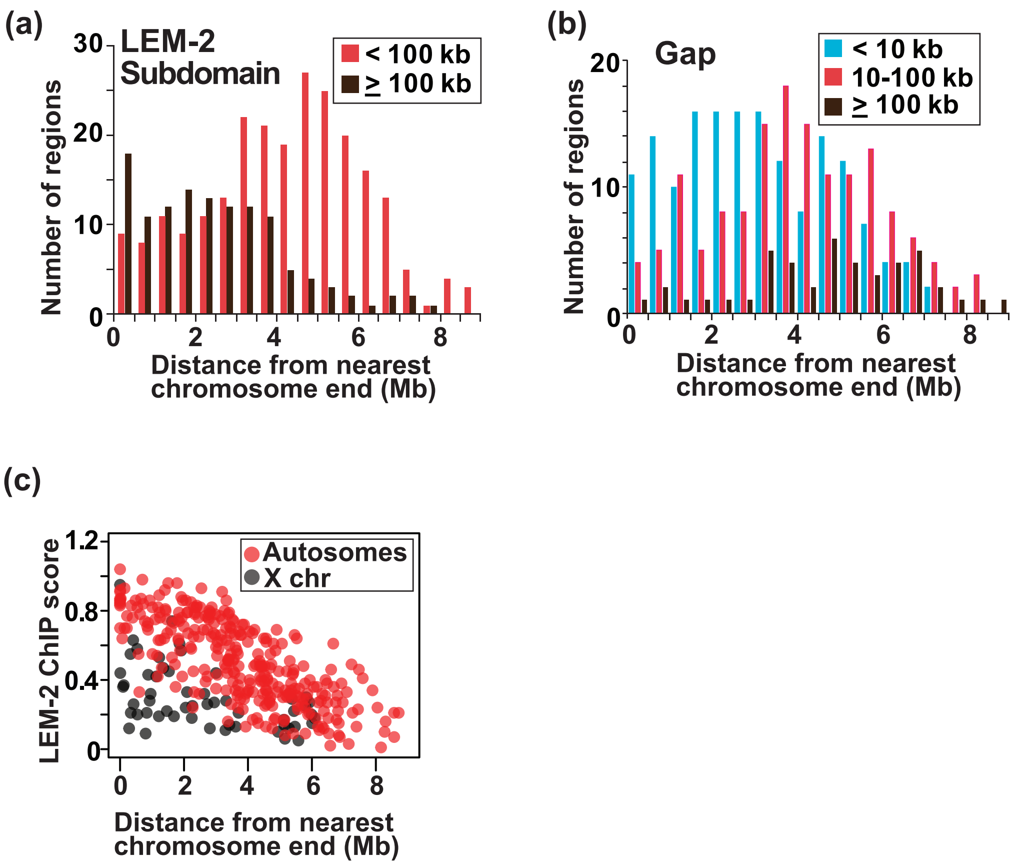

Figure S3. Distribution of LEM-2 subdomains along chromosomes

(a, b) Distribution of LEM-2 subdomains (a) and gaps (b) relative to the distance from the nearest chromosome end. Subdomains or gaps within the indicated size rage were divided into bins every 500 kb from the closest chromosome end, and the counts are plotted. **(c)** Distribution of LEM-2 interaction strength relative to the distance from the closest chromosome end. Each dot represents the average LEM-2 ChIP-chip MA2C score within a subdomain.

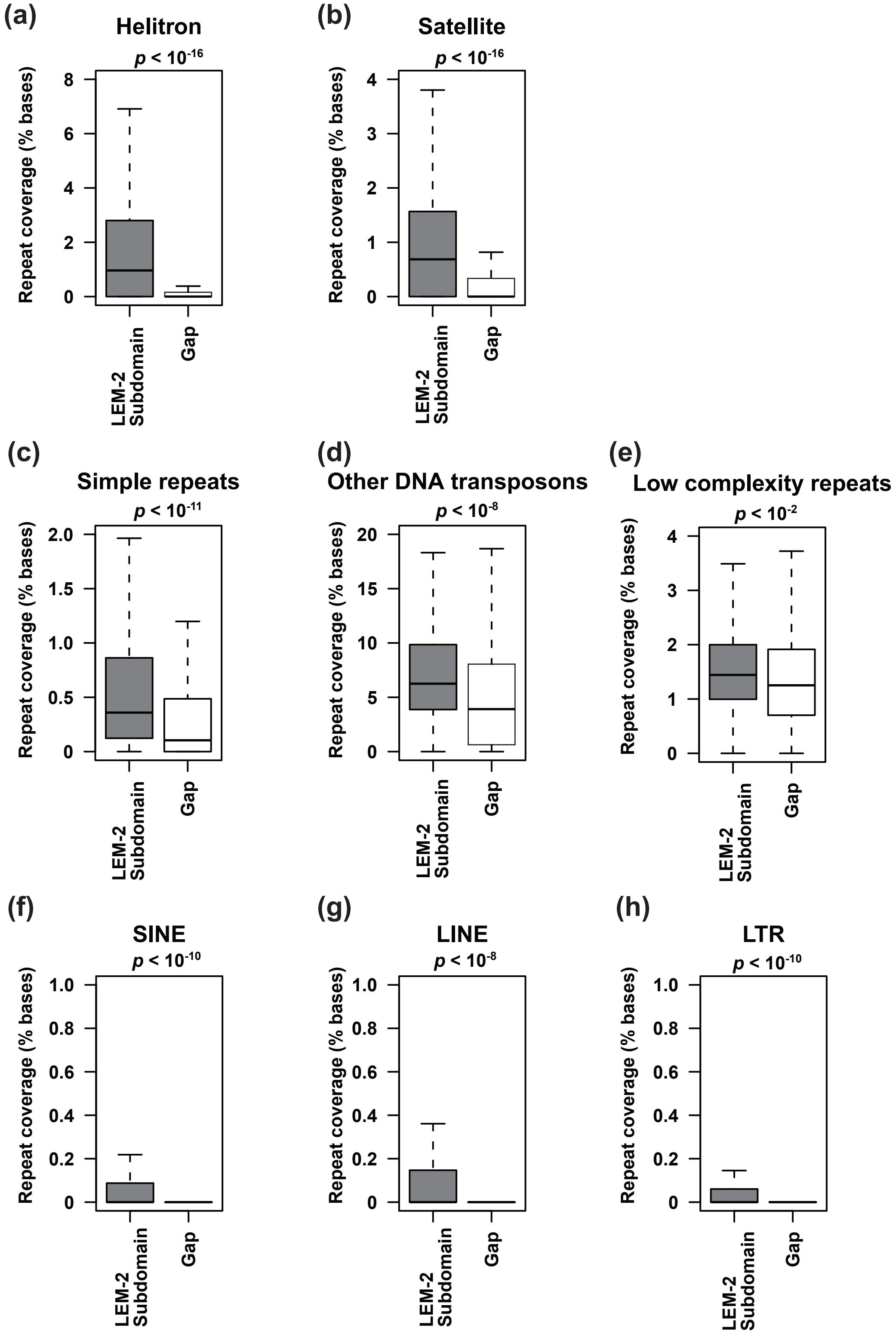

Figure S4. Coverage of different types of repetitive sequences in LEM-2 subdomains and gaps
The percentage of bases covered by different family of repeats (a-h) are plotted. The bottom and top of boxes indicate the 25th and 75th percentiles, respectively, and bands in the boxes indicate medians. Whiskers indicate the lowest or the highest data points within 1.5 x interquartile range from the box. Wilcoxon rank sum test was used for the statistical analysis. Note that gaps in central regions of chromosomes are excluded from this analysis.

Figure S5

Ikegami et al.

(a)

	Position relative to LEM-2 association (this study)	Summary of FISH results in Meister et al. (2010): Peripheral (Within 1/3 of radius from periphery; "Zone 1" in Meister et al.)	Internal (Within 2/3 of radius from center; "Zone 2+3" in Meister et al.)	Figure in Meister et al.
baf-1 gene	Extra large gap (chromosome center)	~30%	~70%	Figure 7C
tbb-1 gene	Small gap	~35%	~65%	Figure 7C
pha-4 gene	LEM-2 subdomain	~80%	~20%	Figure 7D

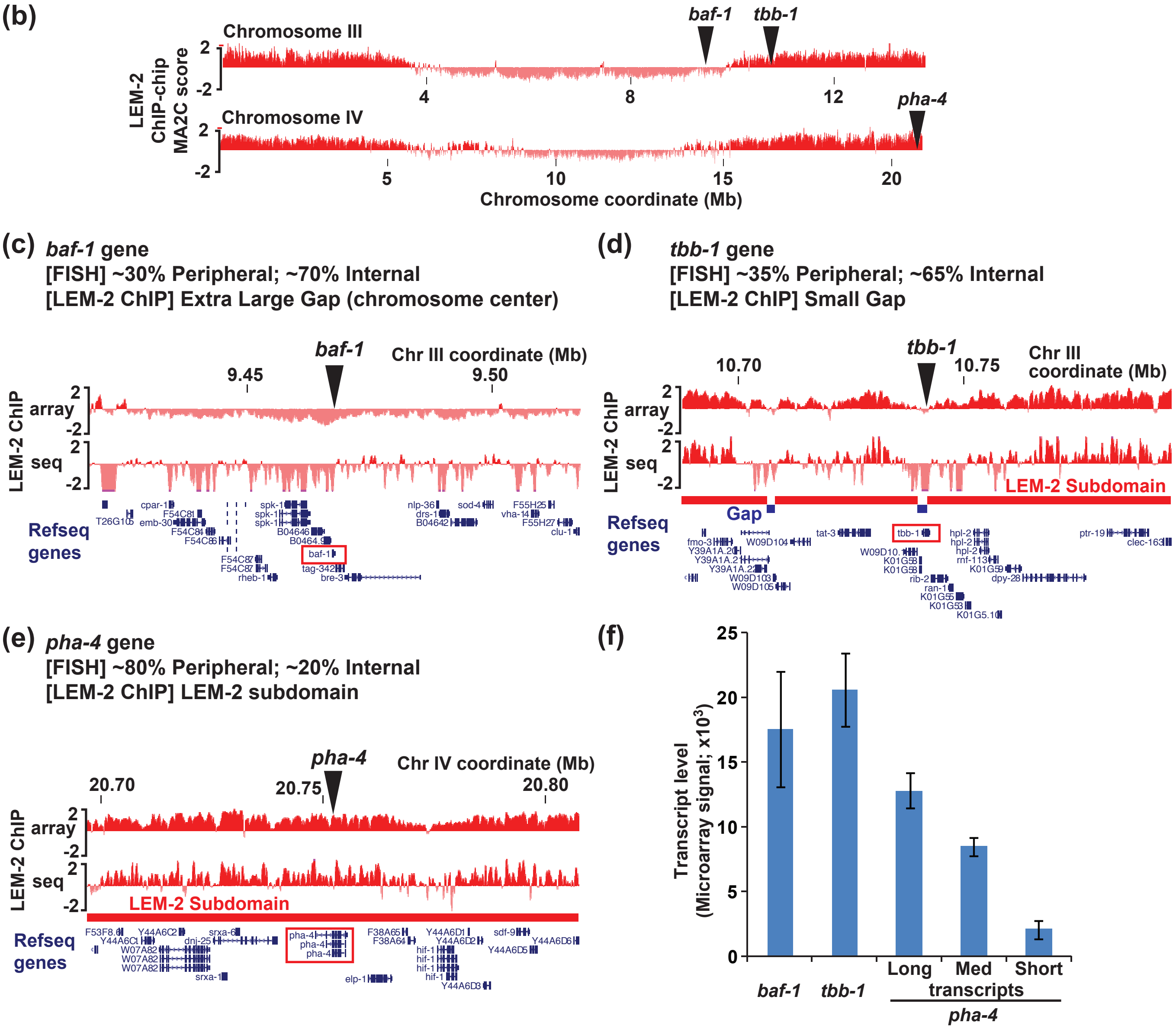

Figure S5. LEM-2 association status of genes for which subnuclear positions were previously determined by fluorescent *in situ* hybridization (FISH)

(a) Summary of LEM-2 ChIP results (this study) and FISH results performed by Meister et al. (2010) for *baf-1*, *tbb-1* and *pha-4* genes. **(b)** Chromosomal positions of *baf-1*, *tbb-1* and *pha-4* genes. **(c-e)** Detailed distribution of LEM-2 ChIP-chip (top) or ChIP-seq (bottom) signals around *baf-1* (c), *tbb-1* (d), and *pha-4* (e) genes. **(f)** Transcript level of *baf-1*, *tbb-1* and *pha-4* genes in mixed-stage embryos determined by expression microarray (Material and methods). Error bars indicate standard deviation among 4 biological replicates.

(a) N2 (Normal karyotype)

(b) mnT12 (X;IV fusion)

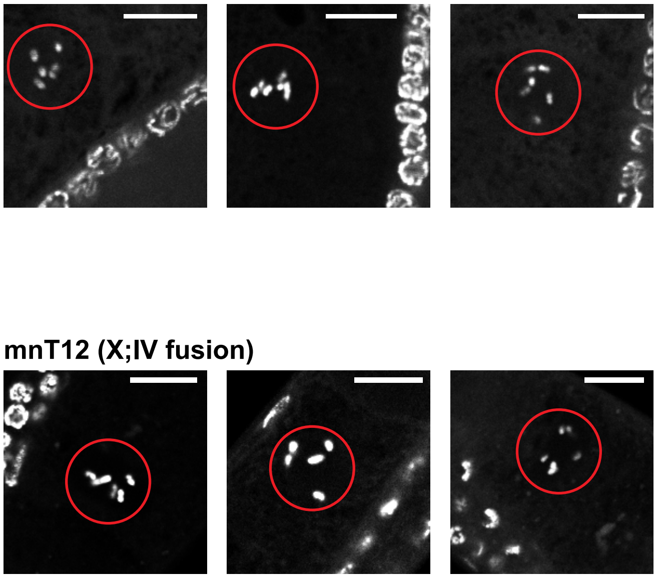

Figure S6. Validation of a strain with a fusion chromosome

(a) Wild-type (N2) chromosomes at diakinesis of meiotic prophase in oocytes. The circles enclose six bivalents of chromosomes (5 autosomes and X chromosome). Three different worms are shown. Chromosomes were stained by DAPI. **(b)** Same as (a), but in the X;IV fusion strain (mnT12). This strain contains five bivalents of chromosomes (4 autosomes and the fusion chromosome). Bars at right upper corners indicate 10 μm.

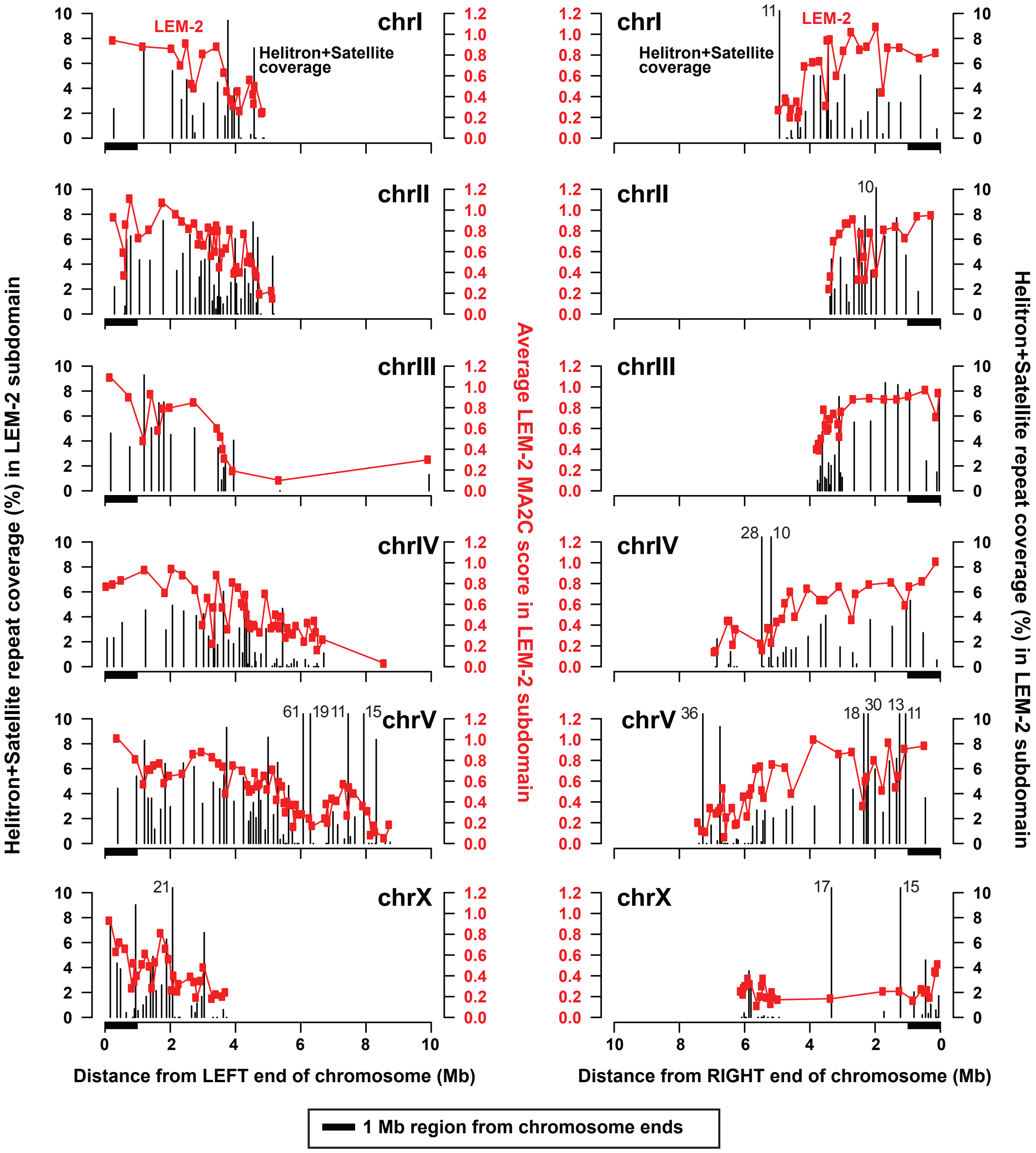

Figure S7. Coverage of helitrons and satellite repeats in LEM-2 subdomains along chromosomes
Black bars indicate percentages of bases covered with helitrons or satellite repeats in LEM-2 subdomains. Red lines indicate average LEM-2 ChIP-chip values in LEM-2 subdomains. Each data point is designated by the central coordinate of the LEM-2 subdomain. The x-axis represents distance from the end of each chromosome. Repeat coverage that exceeds the y-axis range is indicated by the number.

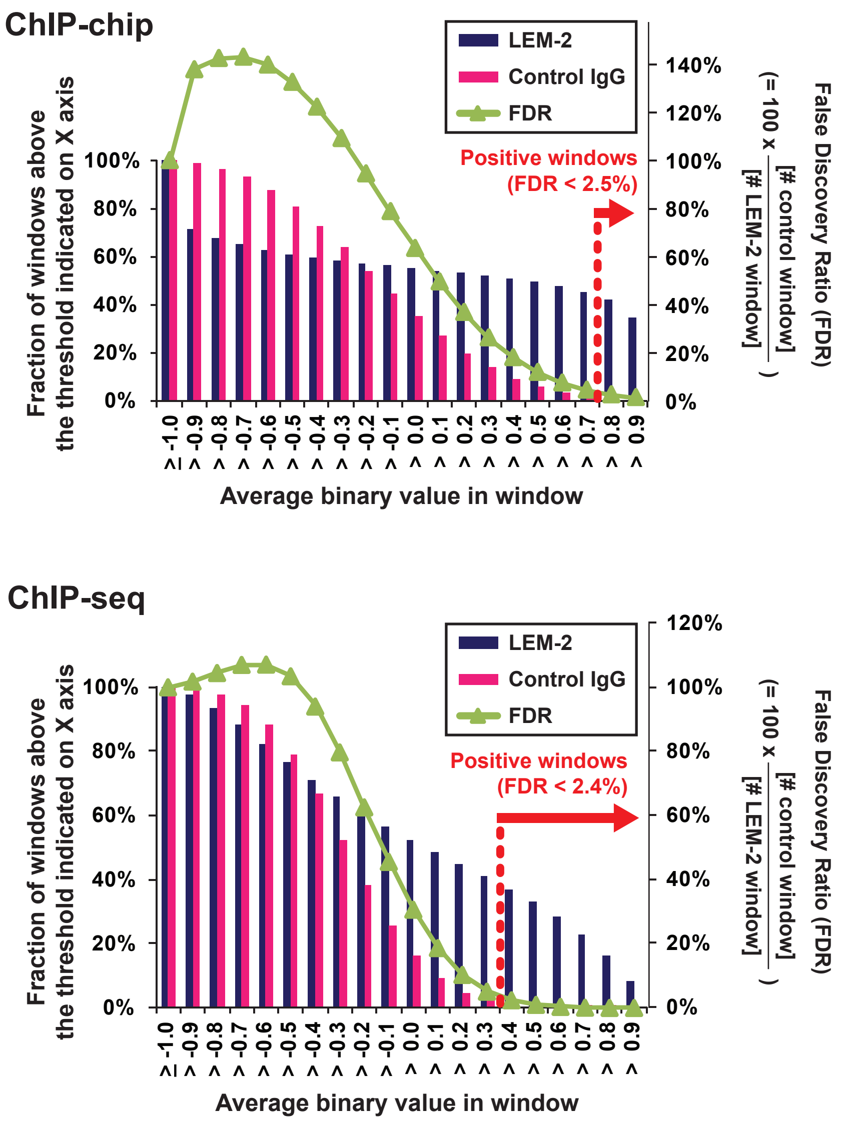

Figure S8. LEM-2 subdomain calling

The x axis bins 10-kb windows in ChIP-chip (200 probes) or ChIP-seq according to thresholds for average binary values within windows (Materials and methods). The left y-axis indicates the number of windows in each bin as a proportion of the total number of windows. The right y-axis indicates False Discovery Ratio (FDR) of each bin, which was estimated by the proportion of the number of control IgG windows to the number of LEM-2 windows.
